# Reciprocal relationships between stress and depressive symptoms: the essential role of the nucleus accumbens

**DOI:** 10.1017/S0033291723002866

**Published:** 2023-09-26

**Authors:** Yizhou Ma, Peter Kochunov, Mark D. Kvarta, Tara LeGates, Bhim M. Adhikari, Joshua Chiappelli, Andrew van der Vaart, Eric L. Goldwaser, Heather Bruce, Kathryn S. Hatch, Si Gao, Shuo Chen, Ann Summerfelt, Thomas E. Nichols, L. Elliot Hong

**Affiliations:** 1 Maryland Psychiatric Research Center, Department of Psychiatry, University of Maryland School of Medicine, Baltimore, MD, USA;; 2 Department of Biological Sciences, University of Maryland, Baltimore County, Baltimore, MD, USA;; 3 Department of Psychiatry, Weill Cornell Medical College/New York-Presbyterian Hospital, New York, NY, USA;; 4 School of Medicine, University of California, San Diego, CA, USA; 5 Department of Statistics, Big Data Science Institute, University of Oxford, Oxford, UK

**Keywords:** depressive symptoms, multimodal neuroimaging, nucleus accumbens, reward processing, stress, UK Biobank, ventral striatum

## Abstract

**Background.:**

Stress and depression have a reciprocal relationship, but the neural underpinnings of this reciprocity are unclear. We investigated neuroimaging phenotypes that facilitate the reciprocity between stress and depressive symptoms.

**Methods.:**

In total, 22 195 participants (52.0% females) from the population-based UK Biobank study completed two visits (initial visit: 2006–2010, age = 55.0 ± 7.5 [40–70] years; second visit: 2014–2019; age = 62.7 ± 7.5 [44–80] years). Structural equation modeling was used to examine the longitudinal relationship between self-report stressful life events (SLEs) and depressive symptoms. Cross-sectional data were used to examine the overlap between neuroimaging correlates of SLEs and depressive symptoms on the second visit among 138 multimodal imaging phenotypes.

**Results.:**

Longitudinal data were consistent with significant bidirectional causal relationship between SLEs and depressive symptoms. In cross-sectional analyses, SLEs were significantly associated with lower bilateral nucleus accumbal volume and lower fractional anisotropy of the forceps major. Depressive symptoms were significantly associated with extensive white matter hyperintensities, thinner cortex, lower subcortical volume, and white matter microstructural deficits, mainly in corticostriatal-limbic structures. Lower bilateral nucleus accumbal volume were the only imaging phenotypes with overlapping effects of depressive symptoms and SLEs (*B* = −0.032 to −0.023, *p* = 0.006–0.034). Depressive symptoms and SLEs significantly partially mediated the effects of each other on left and right nucleus accumbens volume (proportion of effects mediated = 12.7–14.3%, *p* < 0.001–*p* = 0.008). For the left nucleus accumbens, post-hoc seed-based analysis showed lower resting-state functional connectivity with the left orbitofrontal cortex (cluster size = 83 voxels, *p* = 5.4 × 10^−5^) in participants with high *v*. no SLEs.

**Conclusions.:**

The nucleus accumbens may play a key role in the reciprocity between stress and depressive symptoms.

## Introduction

Depression is a leading cause of disability worldwide with over 10% of the population experiencing one or more major depressive episodes in their lifetime (Bromet et al., 2011; World Health Organization, 2020). A well-known risk factor for depression is stress: individuals with a history of childhood adversity, stressful life events (SLEs), or trauma are more likely to develop depression, relapse, and become treatment resistant (Amital, Fostick, Silberman, Beckman, & Spivak, 2008; Chapman et al., 2004; Kendler, Karkowski, & Prescott, 1999; Widom, DuMont, & Czaja, 2007). The tie between stress and depression is also reciprocal: individuals with depression are at a higher risk of encountering stress in part due to their experience with depression and its sequelae including functional impairment (Conway, Hammen, & Brennan, 2012; Hammen, 1991, 2006; van Os & Jones, 1999). Despite this bidirectional relationship, the underlying neural substrates remain unclear. Identifying the neural basis of this reciprocal and potentially reinforcing loop may help develop more effective interventions that build resilience against both stress and depression.

Both depression and stress involve neurobiological pathways in the limbic, paralimbic, and prefrontal structures and their orchestrated regulation of stress-related hormones and neurotransmitters (Godoy, Rossignoli, Delfino-Pereira, Garcia-Cairasco, & Umeoka, 2018; Gold, Machado-Vieira, & Pavlatou, 2015; Maletic et al., 2007). Neuroimaging meta-analyses show that both major depression and stress-related conditions (e.g. childhood adversity, post-traumatic stress disorder) are associated with lower hippocampal volume, lower paralimbic cortical thickness or volume, reduced white matter integrity in the corpus callosum, and altered connectivity in frontostriatal, orbitofrontal, and limbic networks (Bao et al., 2021; Calem, Bromis, McGuire, Morgan, & Kempton, 2017; Dennis et al., 2021; Drysdale et al., 2017; Kraynak, Marsland, Hanson, & Gianaros, 2019; Lim, Howells, Radua, & Rubia, 2020; Lim, Radua, & Rubia, 2014; Logue et al., 2018; Schmaal et al., 2017, 2016; van Velzen et al., 2020; Wang et al., 2021). Despite these overlaps, few human studies have directly compared the neural correlates of stress and depression in the same individuals.

In rodents, stress-induced depressive-like behavior has been linked to neurobiological changes in regions such as the hippocampus, amygdala, and ventral striatum (Brenes, Rodríguez, & Fornaguera, 2008; Hollis, Wang, Dietz, Gunjan, & Kabbaj, 2010; Zan et al., 2021). In humans, history of stress is found to independently contribute to hippocampal, prefrontal, temporal, and white matter alterations in depression (Chaney et al., 2014; Frodl, Reinhold, Koutsouleris, Reiser, & Meisenzahl, 2010; Kronmüller et al., 2008; Meinert et al., 2019; Tozzi et al., 2020; van Harmelen et al., 2010; Vythilingam et al., 2002). However, a prevailing focus of existing neurobiological research is the unidirectional effect of stress on depression, while the reverse is over-looked where depression increases the vulnerability to stress. To our knowledge, no studies have attempted to identify overlapping brain regions in stress and depression and their role in the reciprocal stress–depression relationship. In this study, we explored this question in a large sample of community adults. We chose to study depressive symptoms, rather than clinical depression, as it can be measured continuously and fluctuate in individuals regardless of a diagnosis of depression. We hypothesized that SLEs experienced by individuals would predict their depressive symptoms over time and vice versa. We conducted brain-wide association analyses across multimodal neuroimaging phenotypes to capture brain structures shared between SLEs and depressive symptoms. Based on the literature, we predicted that SLEs and depressive symptoms would share neuroimaging correlates in structures including the hippocampus, amygdala, hypothalamus, ventral striatum, and prefrontal cortex (Brenes et al., 2008; Chaney et al., 2014; Frodl et al., 2010; Hollis et al., 2010; Kronmüller et al., 2008; Meinert et al., 2019; Tozzi et al., 2020; van Harmelen et al., 2010; Vythilingam et al., 2002; Zan et al., 2021), which may be the key region(s) to understand their reciprocity.

## Materials and methods

### Participants

In total, 22 195 community adults (52.0% female) in the UK Biobank (UKBB)’s neuroimaging data release version 1.6 were included. We used data from the initial assessment visit (v0, 2006–2010) and the second visit with brain imaging (v2, 2014–2019). The first repeat assessment visit (v1), completed by only 24.5% participants, was not used. Participants’ age was 55.0 ± 7.5 (range 40–70) years on v0 and 62.7 ± 7.5 (44–80) years on v2. Interval between visits was 7.7 ± 1.5 (4–12) years. Behavioral and neuroimaging protocols were previously described (Miller et al., 2016; Sudlow et al., 2015). The UKBB was approved by the North West Multi-center Research Ethics Committee. All participants provided written informed consent. We received approval from the UKBB to access and analyze the data. The authors assert that all procedures contributing to this work comply with the ethical standards of the relevant national and institutional committees on human experimentation and with the Helsinki Declaration of 1975, as revised in 2008.

### Stressful life events (SLEs)

The UKBB does not use a validated inventory for SLEs but includes a screening question on stressful events such as illness, injury, and bereavement. On both visits, participants reported if they experienced the following in the last two years: ‘serious illness, injury, or assault to yourself’, ‘serious illness, injury, or assault of a close relative’, ‘death of a close relative’, ‘death of a spouse or partner’, ‘marital separation/divorce’, and ‘financial difficulties’. Research in the UKBB showed that SLEs measured by this question was significantly positively associated with psycho-pathology and chronic pain (Davis et al., 2020; Macfarlane, Beasley, & Macfarlane, 2014). Following a previous study (Macfarlane et al., 2014), we defined SLEs as the number of events reported, and recoded values larger than 3 as 3 to reduce right skewness. Alternatively, we used log transformation to reduce skewness and reported findings in online Supplementary Results. In total, 97.1% of the participants had data for both visits.

As illustrated in online Supplementary Fig. S1, on both visits, most participants reported no SLEs. On average, around 30% of the participants reported one SLE; 8% reported two SLEs; and 1.5% reported three or more SLEs (3 SLEs: 1.3%, 4 SLEs: 0.2%, 5 SLEs: 0.01%). The two most reported SLEs were ‘death of a close relative’ (around 20%) and ‘serious illness, injury or assault of a close relative’ (around 13%). The least reported SLE was ‘death of a spouse or partner’ (less than 1.5%). On both v0 and v2, more SLEs were associated with younger age (*r* = −0.11 and −0.14, *p* < 2 × 10^−16^), female sex (*B* = 0.08 and 0.10, *p* < 2 × 10^−16^), and non-White British ancestry (*B* = 0.06 and 0.04, *p* = 0.0002 and 0.006).

### Depressive symptoms

The UKBB does not use a validated tool to measure depressive symptoms on each visit. Following a previous study (Arnau-Soler et al., 2019), we measured participants’ depressive symptoms over the preceding 2 weeks with four touchscreen questions. Two questions were from the Patient Health Questionnaire (PHQ)-2 (Kroenke, Spitzer, & Williams, 2003) and measured core depressive symptoms of anhedonia (‘had little interest or pleasure in doing things’) and depressed mood (‘felt down, depressed, or hopeless’). One question was from the PHQ-9 (Spitzer, Kroenke, Williams, & Group, 1999) and measured fatigue (‘felt tired or had little energy’). The last question measured psychomotor agitation (‘felt tense, fidgety, or restless’). On both visits, participants rated their symptoms over the past 2 weeks from ‘not at all’ to ‘nearly every day’ (0–3). Cronbach’s *α* was 0.8 on v0 and 0.78 on v2. Following the previous study (Arnau-Soler et al., 2019), we transformed the sum of depressive symptoms to a four-point scale to correct for right skewness (0 = 0, 1 = 1–2; 2 = 3–5, 3 = 6 or more). Findings when log transformation was used to correct for skewness were included in online Supplementary Results. In total, 88.4% of the participants had data for both visits.

To further examine the validity of the depressive symptoms measure, we compared depressive symptoms across participants with different lifetime history of depression (Smith et al., 2013). On both visits, participants with a history of probable recurrent depression had significantly more depressive symptoms than participants with a history of probable single episode depression, followed by subclinical depression and mood disorder controls, supporting the validity of the depressive symptoms measure. Details of this analysis, including criteria used to determine lifetime history of depression, can be found in the online Supplementary Methods.

Online Supplementary Fig. S3 illustrates the distribution of depressive symptoms across the two visits. On average, around 42% of the participants reported no depressive symptoms; around 34% scored 1; 14% scored 2; and 3% scored 3. On both visits, more depressive symptoms were associated with younger age (*r* = −0.18 and −0.16, *p* < 2 × 10^−16^), female sex (*B* = 0.14 and 0.16, *p* < 2 × 10^−16^), and non-White British ancestry (*B* = 0.04 and 0.06, *p* = 0.04 and 0.007).

### Adverse childhood experiences (ACEs)

As SLEs may be influenced by ACEs (Gheorghe, Li, Gallacher, & Bauermeister, 2021), we considered ACEs reported by a subsample of participants (71.3%). We measured ACEs as the responses to the Childhood Trauma Screener (Glaesmer et al., 2013; Witt et al., 2022), which surveys participants’ experiences of emotional neglect, physical neglect, emotional abuse, physical abuse, and sexual abuse in childhood. We dichotomized each item and summed them to result in total ACEs ranging from 0 to 5 (Gheorghe et al., 2021).

### Multimodal imaging phenotypes

All participants completed multimodal neuroimaging on v2. Given that no neuroimaging was performed at v0, all analyses involving neuroimaging were cross-sectional using v2 data. We examined 138 imaging phenotypes generated by the UKBB (online Supplementary Table S4), including (1) cortical thickness (CT) of 68 regions; (2) intracranial volume; (3) subcortical volume (SV) of 14 structures; (4) total volume of white matter hyperintensities; and (5) weighted-mean fractional anisotropy (FA) and mean diffusivity (MD) of 27 white matter tracts. Details of image acquisition, quality control, and processing were previously described (Alfaro-Almagro et al., 2018; Miller et al., 2016). Briefly, participants completed T1-MPRAGE (voxel size = 1 mm^3^), T2-FLAIR (voxel size = 1.05 × 1 × 1 mm^3^), and diffusion (50 × *b* = 1000 and 2000 s/mm^2^, voxel size = 2mm^3^) magnetic resonance imaging (MRI). CT, SV, and intracranial volume were derived from UKBB’s T1 pipeline (Alfaro-Almagro et al., 2018) and generated with FreeSurfer (Desikan et al., 2006; Fischl et al., 2004) and FSL’s FIRST tool (Patenaude, Smith, Kennedy, & Jenkinson, 2011). White matter hyperintensities were derived from UKBB’s T2 pipeline (Alfaro-Almagro et al., 2018). FA and MD were derived from UKBB’s diffusion pipeline (Alfaro-Almagro et al., 2018) and generated with FSL’s TBSS tool (Smith et al., 2006). For each phenotype, observations outside of six mean absolute deviations from the median were excluded.

### Resting-state functional MRI (rsfMRI)

Participants completed 6 min of rsfMRI on v2 (TR = 735 ms, TE = 39 ms, flip angle = 52°, voxel size = 2.4 mm^3^). Images acquisition, quality control, and processing were previously described (Alfaro-Almagro et al., 2018; Miller et al., 2016), which included artifact removal with FMRIB’s ICA-based X-noiseifier (Beckmann & Smith, 2004; Griffanti et al., 2014; Salimi-Khorshidi et al., 2014). We additionally completed the following in FSL: spatial registration to the 2 mm MNI152 space, high-pass filtering (FWHM = 2355 s), and spatial smoothing (FWHM = 4 mm, iso-tropic Gaussian kernel).

### Statistical analysis

**Figure 1.**
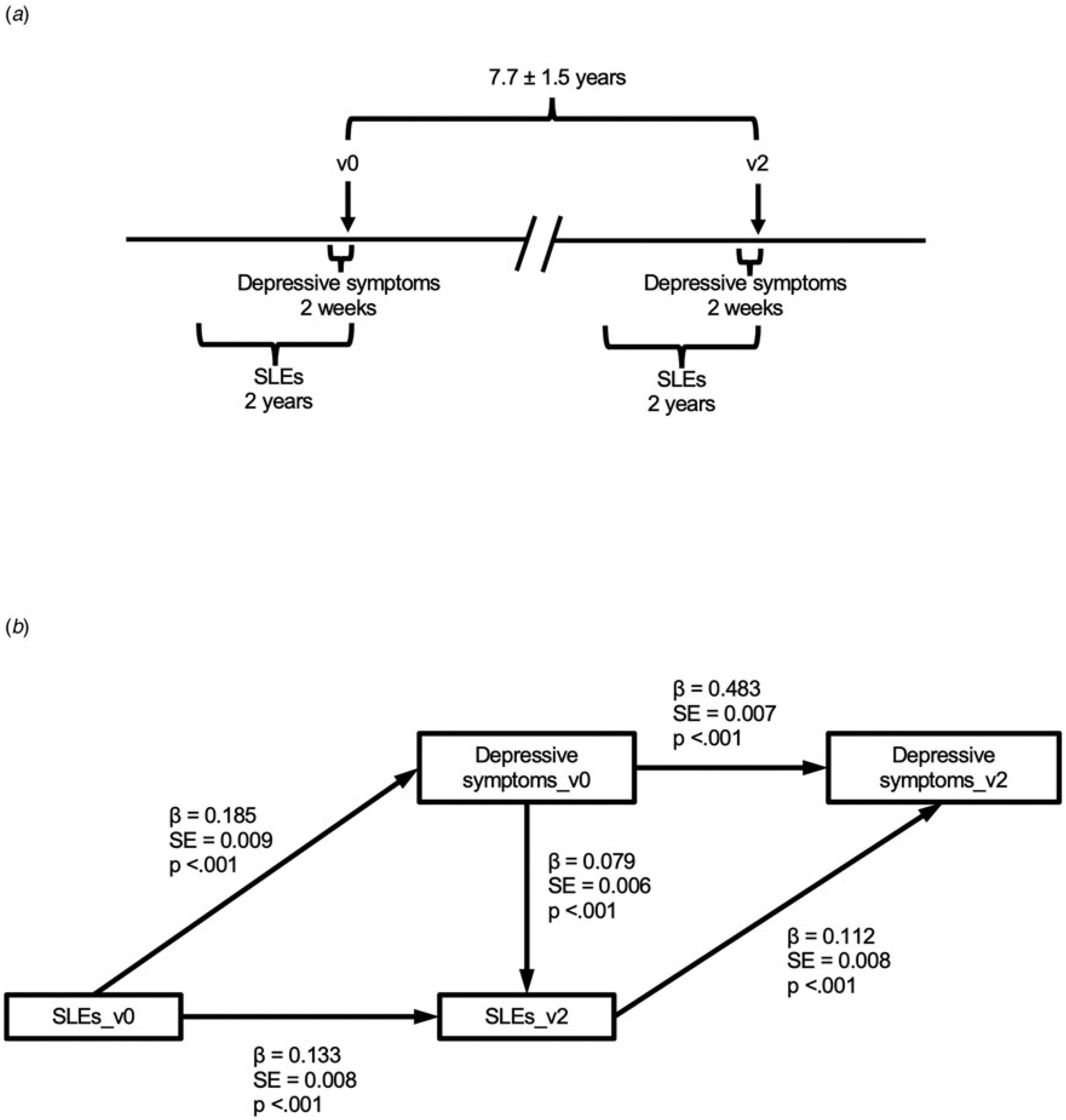
Relationship between SLEs and depressive symptoms based on structural equation modeling. (a) Timeline of repeated assessments across two visits and four time windows. (b) Structural equation model showing bidirectional relationship between depressive symptoms and SLEs. SLEs: stressful life events. *β*, standardized coefficient; S.E., standard error; v0, initial assessment visit; v2, second visit that included brain imaging.

Figure 1a illustrates the data structure used to explore the longitudinal reciprocity between stress and depressive symptoms. We hypothesized that depressive symptoms on v0 would predict more subsequent SLEs when controlling for previous SLEs (here proxied by SLEs within 2 years before v2 and v0, respectively). We also hypothesized that SLEs within 2 years before v2 would predict more subsequent depressive symptoms when controlling for previous depressive symptoms (here proxied by depressive symptoms within 2 weeks before v2 and v0, respectively). We implemented longitudinal structural equation modeling using the lavaan package (Rosseel, 2012) in R (R Core Team, 2019), with maximum likelihood estimation with robust standard errors and Satorra-Bentler scaled test statistics.

We identified imaging phenotypes associated with SLEs on v2 by regressing each standardized imaging phenotype on SLEs in R, correcting for 138 phenotypes with false discovery rate (FDR) *q* < 0.05 (Benjamini & Hochberg, 2000). The same analysis was repeated for depressive symptoms. We then identified which structure(s) were significantly associated with both SLEs and depressive symptoms.

To examine whether imaging phenotypes significantly associated with both SLEs and depressive symptoms were implicated in their reciprocity, we tested two mediation models using v2 data. One model tested the effect of depressive symptoms mediating the relationship between SLEs and imaging phenotypes. The other tested the effect of SLEs mediating the relationship between depressive symptoms and imaging phenotypes. We used the mediation package (Tingley, Yamamoto, Hirose, Keele, & Imai, 2014) in R with 1000 nonparametric bootstraps.

We further performed post-hoc resting-state functional connectivity (rsFC) analysis on v2 using structures significantly associated with both SLEs and depressive symptoms as seeds. RsFC was the Fisher’s *z* transformed Pearson’s correlation between a voxel’s timeseries and a seed’s mean timeseries. To reduce computational and storage burden, we selected a subsample *a priori* (*N* = 1932) to form a balanced 2 × 2 factorial design between SLEs and depressive symptoms. Specifically, we selected participants with high SLEs (HS, when SLEs ≥ 2) *v*. no SLEs (NS) and high depressive symptoms (HD, when depressive symptoms ≥ 2) *v*. no depressive symptoms (ND). Among participants who completed rsfMRI, 483 were HS and ND, 509 HS and HD, 1498 NS and HD, and 5795 NS and ND. The final subsample thus included 483 participants from each group, with NS and HD and NS and ND one-on-one matched to HS and ND for age (±3 years) and sex. Because the HS and HD pool was small, we could not fully match this group to HS and ND on age (HS and ND = 60.9 ± 6.7 years, HS and HD = 58.0 ± 6.5 years, *p* = 8.1 × 10^−12^) and sex (HS and ND = 59.0% female, HS and HD = 67.7%, *p* = 0.006), and we included age and sex as covariates. We set threshold contrasts for SLEs and depressive symptoms with the cluster command in FSL (voxelwise threshold: *p* = 0.001, clusterwise threshold: *p* = 0.05). A post-hoc power analysis suggested that with the current sample size, the voxelwise power to detect the main effect of SLEs or depressive symptoms on rsFC when effect size was small (i.e. Cohen’s *f* = 0.1, equivalent to *d* = 0.2) was 0.86.

Table 1 summarizes the participants used in each analysis. All statistical analyses controlled for age, age^2^, sex, and White British ancestry. Models involving imaging also controlled for handedness (left/right/mixed), scanning site, and *x*, *y*, and *z* head positions in the scanner. Additionally, CT analysis controlled for whether T2_FLAIR was used with T1 in FreeSurfer preprocessing; SV analysis controlled for intracranial volume; rsFC analysis controlled for head motion. See details of covariates in online Supplementary Table S4.

## Results

### Relationship between SLEs and depressive symptoms

Structural equation modeling revealed a significant bidirectional relationship between SLEs and depressive symptoms (Fig. 1b). Controlling for SLEs reported on v0, depressive symptoms on v0 were positively associated with SLEs reported on v2 (*β* = 0.079, *p* < 0.001), suggesting that more severe depression predicted more future SLEs. Conversely, controlling for depressive symptoms on v0, SLEs reported on v2 were positively associated with depressive symptoms on v2 (*β* = 0.112, *p* < 0.001), suggesting that past SLEs predicted depressive symptoms. An alternative model additionally supported a direct effect of SLEs reported on v0 on depressive symptoms on v2, consistent with lasting effects of SLEs on depressive symptoms (online Supplementary Fig. S4).

### SLEs, depressive symptoms, and imaging phenotypes

**Figure 2.**
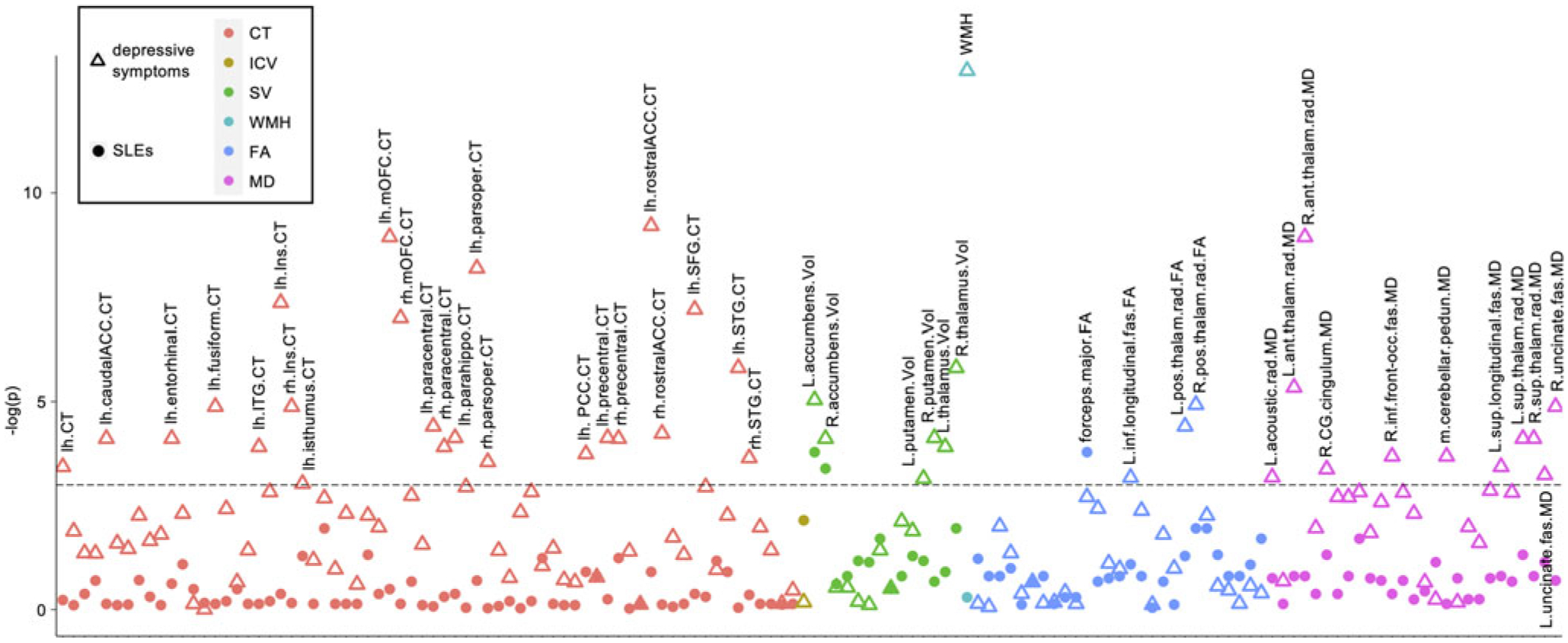
Imaging phenotypes associates of depressive symptoms and SLEs. All *p* values were false discovery rate (FDR) corrected. Dashed line: −log(*p*) corresponding to corrected *p* = 0.05. CT, cortical thickness; ICV, intracranial volume; SV, subcortical volume; WMH, white matter hyperintensities; FA, fractional anisotropy; MD, mean diffusivity; SLEs, stressful life events. For names of the imaging phenotypes, see online Supplementary Table S4.

More SLEs were associated with three imaging phenotypes (Fig. 2, online Supplementary Table S5), including lower forceps major FA (*B* = −0.039, *p*_FDR_ = 0.023) and lower bilateral nucleus accumbens volume (left: *B* = −0.032, *p*_FDR_ = 0.023; right: *B* = −0.030, *p*_FDR_ = 0.034; *B*: differences in standardized imaging phenotypes when SLEs increased by 1). As expected, SLEs and ACEs were significantly positively correlated (*r* = 0.074, *p* < 2 × 10^−16^). Among the three imaging phenotypes associated with SLEs, more ACEs were only associated with lower forceps major FA (*B* = −0.024, *p* = 0.0007), not bilateral nucleus accumbens volume (left: *B* = 0.0002, *p* = 0.97, right: *B* = −0.006, *p* = 0.35). After controlling for ACEs, the above effects of SLEs remained nominally significant but did not survive *FDR* correction (forceps major FA: *B* = −0.037, *p* = 0.003; nucleus accumbens volume left: *B* = −0.028, *p* = 0.008, right: *B* = −0.025, *p* = 0.019). Controlling for ACEs led to a reduction of the absolute B values by 5.7, 12.0, and 16.8%, respectively, which was expected as more ACEs are known to be linked to more SLEs in adulthood (Halonen et al., 2017) that is also supported by their significant correlation in this cohort.

Higher depressive symptoms were associated with 44 imaging phenotypes including (1) thinner cortex in 23 regions with the strongest effects in the left rostral anterior cingulate and medial orbitofrontal cortices; (2) smaller volume in six subcortical structures with the strongest effects in bilateral nucleus accumbens; (3) higher MD in 11 tracts with the strongest effects in the bilateral anterior thalamic radiation; (4) lower FA in three tracts with the strongest effects in the bilateral posterior thalamic radiation; and (5) higher total white matter hyperintensities (Fig. 2, online Supplementary Table S5).

Lower bilateral nucleus accumbens volumes were the only imaging phenotypes significantly associated with both depressive symptoms (left: *B* = −0.026, *p*_FDR_ = 0.006; right: *B* = −0.023, *p*_FDR_ = 0.016) and SLEs (see above). This remained true when using log transformation instead of the recoding method to correct for right skewness in SLEs and depressive symptoms (online Supplementary Results, Table S6).

### Mediation effects

**Figure 3.**
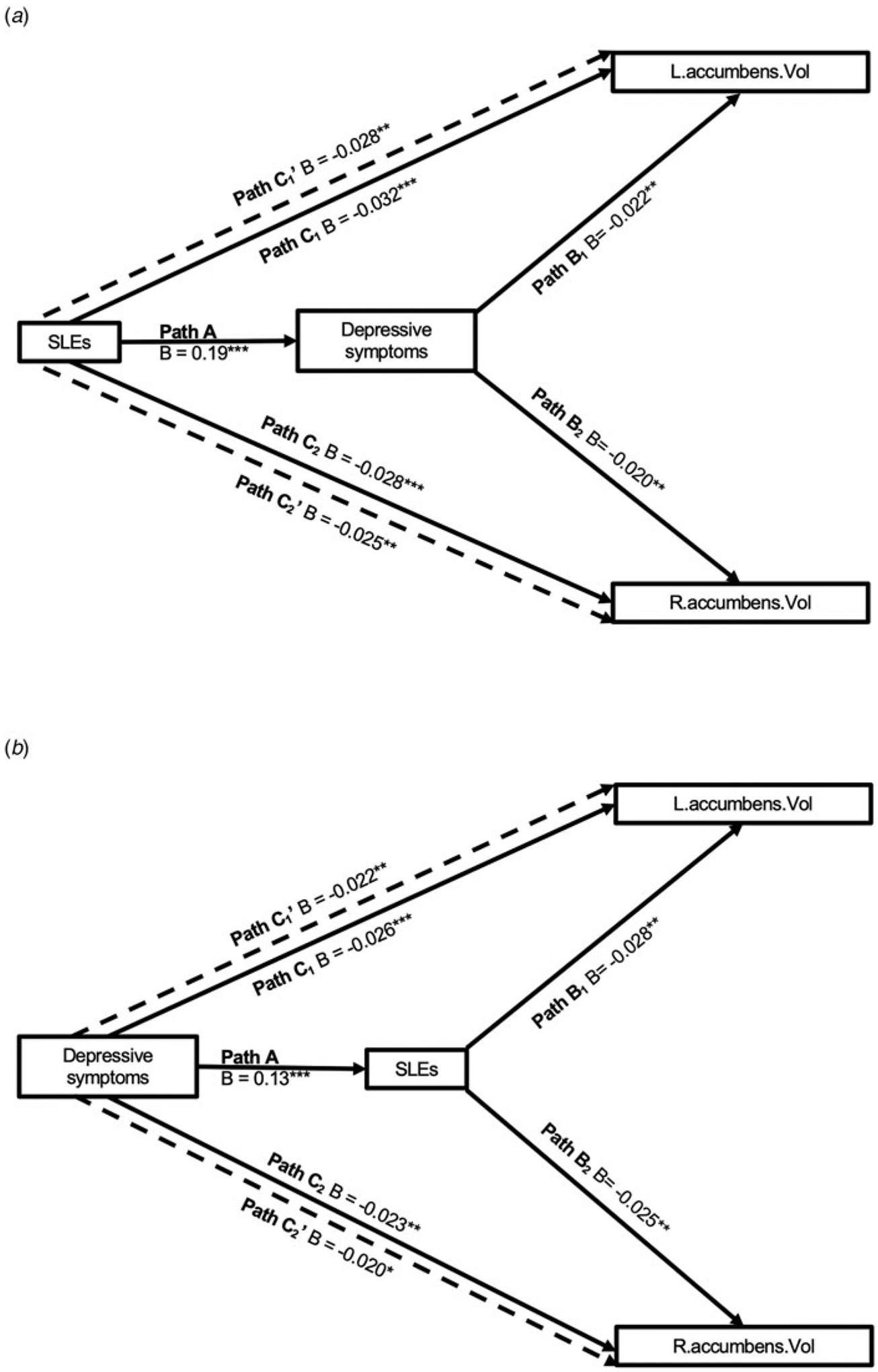
Mediation effects. (a) Depressive symptoms significantly mediated the relationship between SLEs and bilateral nucleus accumbens volume. (b) SLEs significantly mediated the relationship between depressive symptoms and bilateral nucleus accumbens volume. Depressive symptoms and SLEs were measured at v2. All paths controlled for age, age^2^, sex, and White British ancestry. Paths involving the nucleus accumbens volume additionally controlled for handedness, scanning site, *x*, *y*, and *z* head positions in the scanner, and intracranial volume. SLEs: stressful life events. L, left; R, right; Vol, volume. *B* values may be slightly different from those reported in online Supplementary Table S5 due to slightly different numbers of cases with complete data.

Depressive symptoms partially and significantly mediated the relationship between SLEs and bilateral nucleus accumbens volume (left: indirect effect = −0.004, *p* < 0.001, 95% confidence interval [CI] −0.007 to −0.001, 12.8% of the total effect was mediated; right: indirect effect = −0.004, *p* = 0.012, 95% CI −0.006 to −0.001, 12.7% mediated) (Fig. 3a). Conversely, SLEs also partially and significantly mediated the relationship between depressive symptoms and bilateral nucleus accumbens volume (left: indirect effect = −0.004, *p* = 0.002, 95% CI −0.006 to −0.001, 14.3% mediated; right: indirect effect = −0.003, *p* = 0.008, 95% CI −0.006 to −0.001, 14.3% mediated) (Fig. 3b). The mediation effects in both directions accounted for similar proportion of the total effects.

Ideally, the model in Fig. 3b would use depressive symptoms reported immediately prior to SLEs, while we used depressive symptoms on v2 as a proxy (Chiappelli, Nugent, Thangavelu, Searcy, & Hong, 2014). However, when using depressive symptoms on v0 instead, the mediation effects remained largely the same (left: indirect effect = −0.003, *p* < 0.001, 95% CI −0.005 to −0.001, 14.5% mediated; right: indirect effect = −0.003, *p* = 0.004, 95% CI −0.004 to −0.001, 11.5% mediated), supporting that longitudinally experienced depressive symptoms may have influenced the nucleus accumbens via increased occurrences of SLEs. To further test this hypothesis, we tentatively categorized SLEs based on their likelihood to be a consequence of depressive symptoms (Brown & Harris, 1978; Paykel, 1987). Independent SLEs (iSLEs), or events that are more likely independent from depressive symptoms, were ‘serious illness, injury, or assault of a close relative’, ‘death of a close relative’, and ‘death of a spouse or partner’. Dependent SLEs (dSLEs), or events that are more likely partly dependent on depressive symptoms, were ‘marital separation/divorce’ and ‘financial difficulties’. ‘Serious illness, injury, or assault to yourself’ was excluded as it could be either an iSLE or dSLE. Consistent with our hypothesis, dSLEs significantly mediated the relationship between depressive symptoms and nucleus accumbens volume (left: indirect effect = −0.003, *p* = 0.05, 95% CI −0.005 to −0.001, 10.2% mediated; right: indirect effect = −0.003, *p* = 0.018, 95% CI −0.006 to −0.001, 13.3% mediated), but not iSLEs (left: *p* = 0.130; right: *p* = 0.360). However, caution must be taken to interpret this finding given the putative classification of SLEs.

### Nucleus accumbens functional connectivity

**Figure 4.**
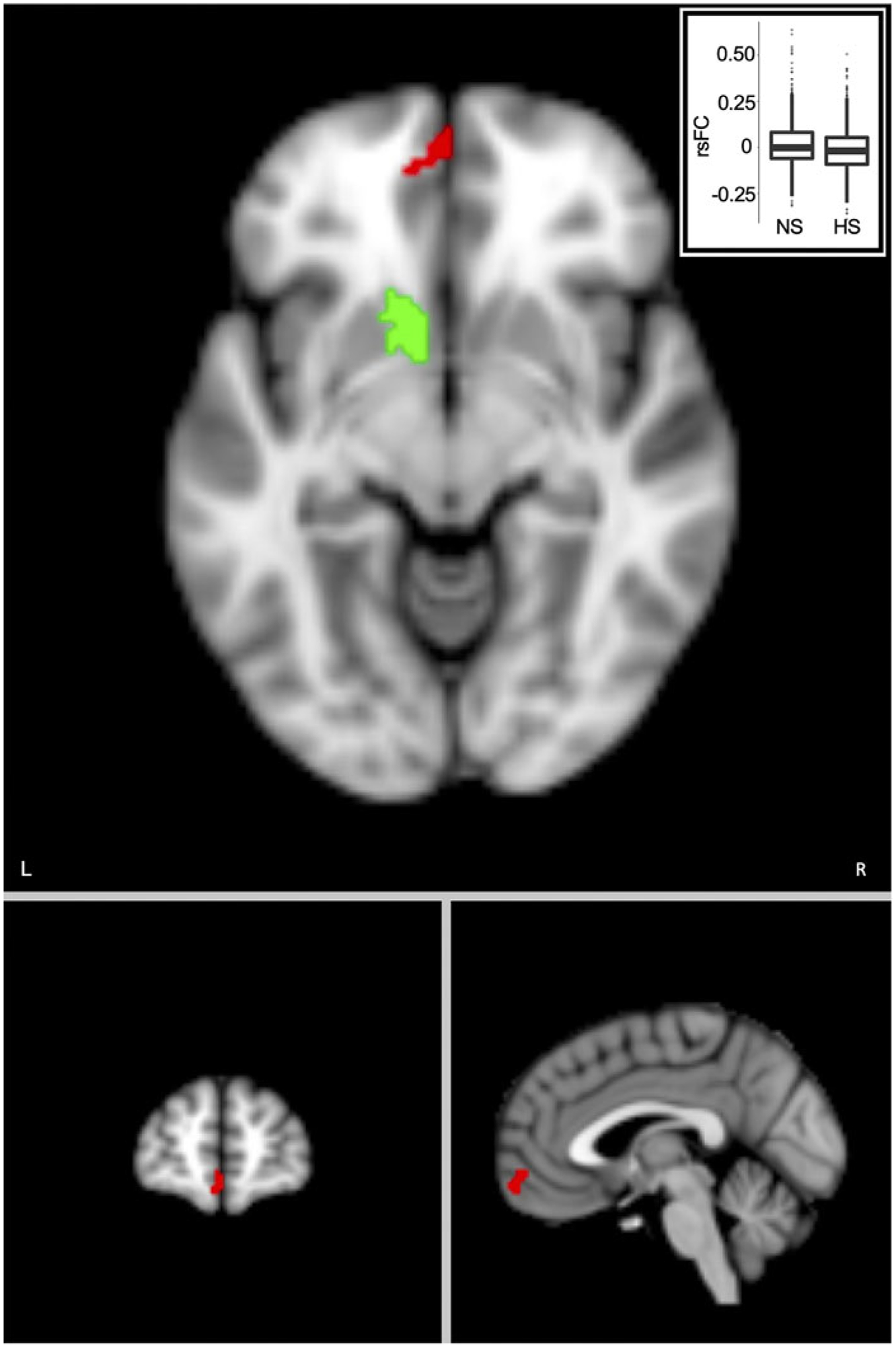
SLEs were associated with lower resting-state functional connectivity (rsFC) between the left nucleus accumbens region-of-interest (green) and the left orbitofrontal cortex (red). Voxel-wise threshold, *p* = 0.001, cluster-wise threshold, *p* = 5.4 × 10^−5^. Imbedded box is the plot comparing rsFC in the high SLEs (HS) and no SLEs (NS) groups. RsFC values are residuals after regressing out age, age^2^, sex, and White British ancestry, handedness, scanning site, *x*, *y*, and *z* head positions in the scanner, and head motion. L, left; R, right; SLE, stressful life event.

Post-hoc rsFC analysis revealed significantly lower rsFC in HS than NS between the left nucleus accumbens seed and a cluster in the left orbitofrontal cortex (OFC) (Fig. 4; cluster size = 83, clusterwise *p* = 5.4 × 10^−5^, peak *F* value = 21.34, MNI coordinate = [−6, 62, −12]). No significant differences were found between HD and ND or for the right nucleus accumbens seed.

### Replication after excluding neurological cases

Finally, we repeated our analyses after excluding participants with neurological conditions (*N* = 448, 2.0% of the total sample; online Supplementary Table S7). Bilateral nucleus accumbens volume remained the only imaging phenotypes significantly and negatively associated with both SLEs and depressive symptoms (online Supplementary Table S8). Conclusions from the rsFC and mediation analyses were not affected.

## Discussion

We examined the brain structures implicated in the reciprocal relationship between stress and depressive symptoms by studying SLEs and depressive symptoms in a large community sample. We replicated the bidirectional relationship between SLEs and depressive symptoms reported by prior studies (Amital et al., 2008; Chapman et al., 2004; Conway et al., 2012; Hammen, 1991; Kendler et al., 1999; van Os & Jones, 1999; Widom et al., 2007). Neuroimaging correlates of SLEs included three structures and functional connectivity between the left nucleus accumbens and the left OFC. Neuroimaging correlates of depressive symptoms included widespread morphological and white matter microstructural changes. Bilateral nucleus accumbens were the only regions with overlapping effects of SLEs and depressive symptoms, and SLEs and depressive symptoms partially mediated the effect of each other on nucleus accumbens volume. The nucleus accumbens may be a neurobiological nexus for the bidirectional relationship between stress and depressive symptoms.

Stress exposure has long been recognized as a risk factor for depression (Amital et al., 2008; Chapman et al., 2004; Kendler et al., 1999; Widom et al., 2007). Reciprocally, the stress generation hypothesis posits that depressive symptoms put an individual at a higher risk for stressful experiences due to depression-related symptoms, behaviors, characteristics, and social context (Hammen, 1991, 2006). Structural equation modeling of longitudinal data was consistent with this reciprocity between SLEs and depressive symptoms. Bidirectional causal relationship is difficult to test because the proposed causal event, by definition, can be affected by the proposed consequence. Using a longitudinal design allowed this reciprocity to be examined.

We found SLEs to be associated with lower white matter integrity in the forceps major, a tract that involves the splenium part of the corpus callosum. Disruptions in the macro- and microstructure of the corpus callosum are well documented in stress and trauma (De Bellis & Keshavan, 2003; De Bellis et al., 2002; Dennis et al., 2021; Jensen et al., 2018). One mechanism can be stress-induced glucocorticoid increase that affects myelination (Huang, Harper, Evans, Newnham, & Dunlop, 2001). We also found SLEs to be associated with weaker rsFC between the left nucleus accumbens and the left OFC, an area central to emotion regulation and decision making (Bechara, Damasio, & Damasio, 2000). OFC alteration has been previously reported in stress (Hanson et al., 2010; Muhammad, Carroll, & Kolb, 2012) and reduced coordination between the OFC and the nucleus accumbens may reflect impaired self-regulation after stress exposure (Meyer & Bucci, 2016).

We replicated widespread alterations associated with depressive symptoms reported by previous studies (Milak et al., 2005; Schmaal et al., 2017, 2016; Shen et al., 2020; van Velzen et al., 2020). Some discrepancies, such as significantly lower hippocampal volume in recurrent depression (Schmaal et al., 2016) but not in the current sample, may be explained by our focus on depression symptomatology as opposed to diagnosis in an older, mostly subclinical population. Most of the imaging associates of depressive symptoms were not significantly associated with SLEs, which is expected as depressive symptoms can result from various factors other than SLEs (Saveanu & Nemeroff, 2012). One caveat though is that the brief UKBB SLEs assessment could not have captured all sources of stress, and additional brain correlates of stress may not have been discovered.

Animal models of post-traumatic stress disorder and major depression overlap extensively in hippocampus, ventral striatum, PFC, and hypothalamic-pituitary-adrenal axis pathology (Ploski & Vaidya, 2021). Here, we found that the nucleus accumbens was the only brain region associated with both SLEs and depressive symptoms. Notably, both the left and right nucleus accumbens showed overlapping effects of SLEs and depressive symptoms after multiple comparison correction, suggesting that this finding is unlikely fortuitous. Moreover, SLEs partially mediated the effect of depressive symptoms on bilateral nucleus accumbens volume and vice versa. While neuroimaging studies have shown that the brain abnormalities in depression can be attributed to previous history of stress (Meinert et al., 2019; Vythilingam et al., 2002), to our knowledge this is the first study to show the reverse, i.e. the neural associates of stress may be accounted for by preexisting depressive symptoms. Furthermore, we show that the mediation effect of SLEs was stable using depressive symptoms measured across two visits several years apart and was only significant for SLEs that are more likely influenced by depressive symptoms (i.e. dSLEs). Thus, our results are consistent with the stress generation theory of depression.

There are strong neurobiological and psychopathological bases for why the nucleus accumbens stood out as a brain structure involved in stress, depressive symptoms, and their reciprocity. The nucleus accumbens has been associated with stress and depression in humans (Edmiston et al., 2011; Gheorghe et al., 2021; Liu et al., 2021; Wacker, Dillon, & Pizzagalli, 2009; Walsh et al., 2014; Whittle et al., 2014). It plays a central role in the brain’s mesolimbic neurotransmission pathways (Baik, 2020; Fox & Lobo, 2019) by receiving dopaminergic, GABAergic, and glutamatergic projections from the ventral tegmentum, amygdala, and other areas for salience, reward, and punishment processing (Bongioanni et al., 2021; Kohls et al., 2013; Lowes et al., 2021). It is connected to essentially all limbic areas for emotional/motivational responses (Floresco, 2015; Saddoris, Cacciapaglia, Wightman, & Carelli, 2015). The nucleus accumbens is therefore a hub for converging neural processes that regulate stress response and depression formation and the impact of stress (depressive symptoms) on this region increases the vulnerability to the other. Smaller nucleus accumbens volume may be the macroscopic manifestation of these underlying processes.

This study has several limitations. First, the measures of SLEs and depressive symptoms have not been previously validated and are susceptible to recall bias (Monroe, 1982; Monroe, Slavich, Torres, & Gotlib, 2007) (although both have been used in previous studies, see Arnau-Soler et al. [2019]; Davis et al. [2020]; Harshfield et al. [2020]; Lehto et al. [2020]; Macfarlane et al. [2014]). Moreover, given restraints imposed by the UKBB design, we could only probe the longitudinal effects between SLEs and depressive symptoms with proxy measures. These issues, although practical for a very large sample, have likely introduced noises and compromised the size of relationships that could be observed, which cannot be fully compensated for by sample size. Second, while we used longitudinal analysis to probe the relationship between SLEs and depressive symptoms, it cannot be taken as direct evidence of causality. Similarly, our neuroimaging findings used cross-sectional data and can only be interpreted as correlational. Third, the 138 imaging phenotypes surveyed in our analyses were not exhaustive and additional shared neural correlates between SLEs and depressive symptoms may exist, such as rsFC phenotypes. Machine-learning approaches with unsupervised learning may be better suited to identify distributed networks that link SLEs and depression (Genon, Eickhoff, & Kharabian, 2022; Marek et al., 2022). Fourth, the effect sizes were small, and the SLEs findings did not survive FDR correction after controlling for ACEs, suggesting that findings in this study may also be partly attributed to early stressful experiences. Fifth, our analyses did not account for factors such as medication use, psychotherapy, and physical comorbidity. Last, the UKBB sample was predominantly healthy white volunteers (Fry et al., 2017), and generalizability to groups with more pathology and lower socioeconomic status is unclear.

To summarize, a reciprocal relationship between SLEs and depressive symptoms was supported using a longitudinal epidemiological dataset, and this bidirectional susceptibility was found to be associated with the nucleus accumbens. To our knowledge, this is the first study to identify the neural underpinnings of the bidirectional stress–depressive symptoms relationship in humans. Further research may be important for developing new therapeutics to disrupt this relationship and increase resilience for both stress-induced depressive symptoms and depressive symptoms-related stressful experiences.

## Supplementary Material

supplement

## Figures and Tables

**Table 1.** Demographics of the participants included in each analysis

Analyses	Bidirectional relationship, structural phenotypes, mediation	Functional connectivity
*N*	22 189	1932
Age (years, v2)	62.7 ± 7.5	58.8 ± 6.6
% Female	52.0%	65.6%
White British ancestry (%)	86.1%	84.2%
College education	47.7%	48.4%
Household income	2.8 ± 1.1	2.8 ± 1.1

*Note:* Household income: (1) less than £18 000; (2) £18 000–£30 999; (3) £31 000–£51 999; (4) £52 000–£100 000; (5) greater than £ 100 000.

## Data Availability

Data supporting the findings of this study are available upon reasonable request from the corresponding author. Raw data are available through the UK Biobank.
