# Supplementary material for: Reciprocal relationships between stress and depressive symptoms: the essential role of the nucleus accumbens
Source: Psychol Med. Author manuscript; Available in PMC 2024 May 8. (PMC11078439; doi:10.1017/S0033291723002866)

**Supplementary Information**

**Supplementary Methods**

**Depressive symptoms across lifetime depression phenotypes**

To validate the depressive symptoms measure, we explored whether it differed across participants with different levels of lifetime depression. We classified participants based on their lifetime history of depression (probable recurrent depression, probable single episode depression, subclinical depression, and no history of mood disorder). We integrated previously published classification criteria based on participants’ responses to the touchscreen questionnaire at each visit (Smith et al., 2013) with hospital records and self-report diagnoses. Details of the classification are listed in **Table S1**. Notably, none of the classification criteria overlapped with the four items used to measure depressive symptoms. We took a conservative approach to defining individuals with no history of mood disorder so that participants with missing values (and thus preventing a definitive classification) were not classified. For this analysis, we also excluded participants with significant neurological and psychiatric conditions and brain cancer per hospital records (**Table S2**), self-report (**Table S3**), and probable bipolar disorder per (Smith et al., 2013).

Among the 22,195 participants, 5,144 were classified with a lifetime history of probable recurrent depression, 702 probable single episode depression, 5,333 subclinical depression, and 4,526 no history of mood disorder. 6,490 participants were excluded or unclassified. On v0, analysis of variance (ANOVA) and Tukey’s honestly significant difference (HSD) test showed significantly higher depressive symptoms in participants with a lifetime history of probable recurrent depression (1.13 ± 0.90), followed by probable single episode depression (0.95 ± 0.92), subclinical depression (0.70 ± 0.76), and no history of mood disorder (0.55 ± 0.69, *F*(3, 14,833) = 435.9, *p* < 2×10^−16^, all Tukey’s HSD *p* ≤ 1.5×10^−6^). The same was true on v2 (probable recurrent depression: 1.03 ± 0.90, probable single episode depression: 0.84 ± 0.90, subclinical depression: 0.63 ± 0.73, no history of mood disorder: 0.49 ± 0.64, *F*(3, 14,846) = 409, *p* < 2×10^−16^, all Tukey’s HSD *p* ≤ 1.0×10^−7^ , see **Figure S2**). Thus, depressive symptoms differed significantly across subgroups with different lifetime history of depression.

**Supplementary Results**

**Imaging phenotypes and log transformed SLEs and depressive symptoms.**

We alternatively used log transformation to correct for right skewness in SLEs and depressive symptoms (Table S6). We applied logarithm with base 10 after adding a small positive offset (0.1) to SLEs and depressive symptoms to avoid undefined values. More SLEs were associated with lower forceps major FA (B = −0.043, *p*_FDR_ = 0.042), lower bilateral nucleus accumbens volume (left: B = −0.041, *p*_FDR_ = 0.028; right: B = −0.039, *p*_FDR_ = 0.031), and lower intracranial volume (B = −0.032, *p*_FDR_ = 0.042; B: differences in standardized imaging phenotypes when log transformed SLEs increased by 1).

Higher depressive symptoms were associated with 32 imaging phenotypes including thinner cortex, smaller subcortical volume, higher MD, lower FA, and higher total white matter hyper intensities in regions similar to those reported in the main text. Lower bilateral nucleus accumbens volume remained the only imaging phenotypes significantly associated with both depressive symptoms (left: B = −0.030, *p*_FDR_ = 0.014; right: B = −0.027, *p*_FDR_ = 0.030) and SLEs (see above).

**References**

Smith, D. J., Nicholl, B. I., Cullen, B., Martin, D., Ul-Haq, Z., Evans, J., … Pell, J. P. (2013). Prevalence and characteristics of probable major depression and bipolar disorder within UK Biobank: Cross-sectional study of 172,751 participants. *PLoS ONE*, *8*(11), 1–8. https://doi.org/10.1371/journal.pone.0075362

Table S1. Criteria for lifetime depression phenotypes.

| Group | Criteria |
| --- | --- |
| Subclinical | Met criteria in (Smith et al., 2013) for probable single episode or recurrent depression *except for* treatment seeking; OR treatment seeking but did not meet criteria in (Smith et al., 2013) for probable single episode or recurrent depression. |
| Probable single episode depression | Met criteria in (Smith et al., 2013) for probable single episode depression AND no hospital records of recurrent (F33) or persistent mood disorders (F34); OR did not meet criteria in (Smith et al., 2013) for probable single episode but had hospital records of depressive episode (F32). |
| Probable recurrent depression | Met criteria in (Smith et al., 2013) for probable recurrent depression; OR had hospital records of recurrent (F33) or persistent mood disorders (F34). |
| No history of mood disorder | Not treatment seeking; AND did not meet criteria in (Smith et al., 2013) for probable single episode or recurrent depression; AND no hospital records of depression (F32, F33, F34); AND no self-reported depression (“1286” in UK Biobank Field 20002) |

Table S2. Excluded ICD10 diagnoses from lifetime depression phenotypes per hospital records (UK Biobank Field 41270).

| ICD10 code/code blocks | Diagnosis |
| --- | --- |
| C71 | Malignant neoplasm of brain |
| F00 | Dementia in Alzheimer’s disease |
| F01 | Vascular dementia |
| F02 | Dementia in other diseases classified elsewhere |
| F03 | Unspecified dementia |
| F20-F29 | Schizophrenia, schizotypal and delusional disorders |
| F31 | Bipolar affective disorder |
| G00-G09 | Inflammatory diseases of the central nervous system |
| G10 | Huntington’s disease |
| G13 | Systemic atrophies primarily affecting central nervous system in diseases classified elsewhere |
| G30-G32 | Other degenerative diseases of the nervous system |
| G35-G37 | Demyelinating diseases of the central nervous system |
| G40 | Epilepsy |
| G41 | Status epilepticus |
| G80 | Infantile cerebral palsy |

Table S3. Excluded self-report diagnoses from lifetime depression phenotypes (UK Biobank Field 20002).

| Code | Diagnosis |
| --- | --- |
| 1244 | infection of nervous system |
| 1245 | brain abscess/intracranial abscess |
| 1246 | Encephalitis |
| 1247 | Meningitis |
| 1248 | spinal abscess |
| 1249 | cranial nerve problem/palsy |
| 1258 | chronic/degenerative neurological problem |
| 1261 | multiple sclerosis |
| 1262 | parkinsons disease |
| 1263 | dementia/alzheimers/cognitive impairment |
| 1264 | Epilepsy |
| 1289 | Schizophrenia |
| 1291 | mania/bipolar disorder/manic depression |
| 1397 | other demyelinating disease (not multiple sclerosis) |
| 1433 | cerebral palsy |
| 1526 | polio / poliomyelitis |

Table S7. Neurological conditions excluded from sensitivity analyses

| **ICD 10 code** | **Description** |
| --- | --- |
| C70 | malignant neoplasms of meninges |
| C71 | malignant neoplasms of brain |
| C72 | malignant neoplasm of spinal cord, cranial nerves and other parts of central nervous system |
| F0 | Organic, including symptomatic, mental disorders |
| G0 | inflammatory diseases of the central nervous system |
| G20 | Parkinson’s disease |
| G30-32 | Other degenerative diseases of the nervous system |
| G35-37 | Demyelinating diseases of the central nervous system |
| G40 | epilepsy |
| I60 | subarachnoid haemorrhage |
| I61 | intracerebral haemorrhage |
| I63 | Cerebral infarction |
| Q0 | congenital malformations of the nervous system |
| R90 | abnormal findings on diagnostic imaging of the central nervous system |
| R940 | abnormal results of function studies of the central nervous system |
| S04 | Injury of cranial nerves |
| S06 | Intracranial injury |
| S07 | Crushing injury of head |
| S08 | Traumatic amputation of part of head |
| S09 | Other and unspecified injuries of head |

**Supplementary Figure Legends**

**Figure S1**. Distribution (a) and prevalence (b) of SLEs on v0 and v2. SLEs: stressful life events. v0: initial assessment visit. v2: imaging visit. SLEs larger than 3 were recoded as 3 to reduce right skewness.

**Figure S2.** Group differences in depressive symptoms across lifetime depression phenotypes. v0: initial assessment visit. v2: imaging visit. Error bars represent standard deviation. ***p < 0.001.

**Figure S3**. Distribution of depressive symptoms on v0 and v2. v0: initial assessment visit. v2: imaging visit. Raw scores of depressive symptoms were transformed to a 4-point scale to correct for right skewness (0 = 0, 1 = 1-2; 2 = 3-5, 3 = 6 or more).

**Figure S4.** Alternative structural equation model showing bidirectional relationship between depressive symptoms and SLEs and a direct effect of SLEs reported on v0 on depressive symptoms on v2. SLEs: stressful life events. SE: standard error. v0: initial assessment visit. v2: imaging visit.

Figure S1

**
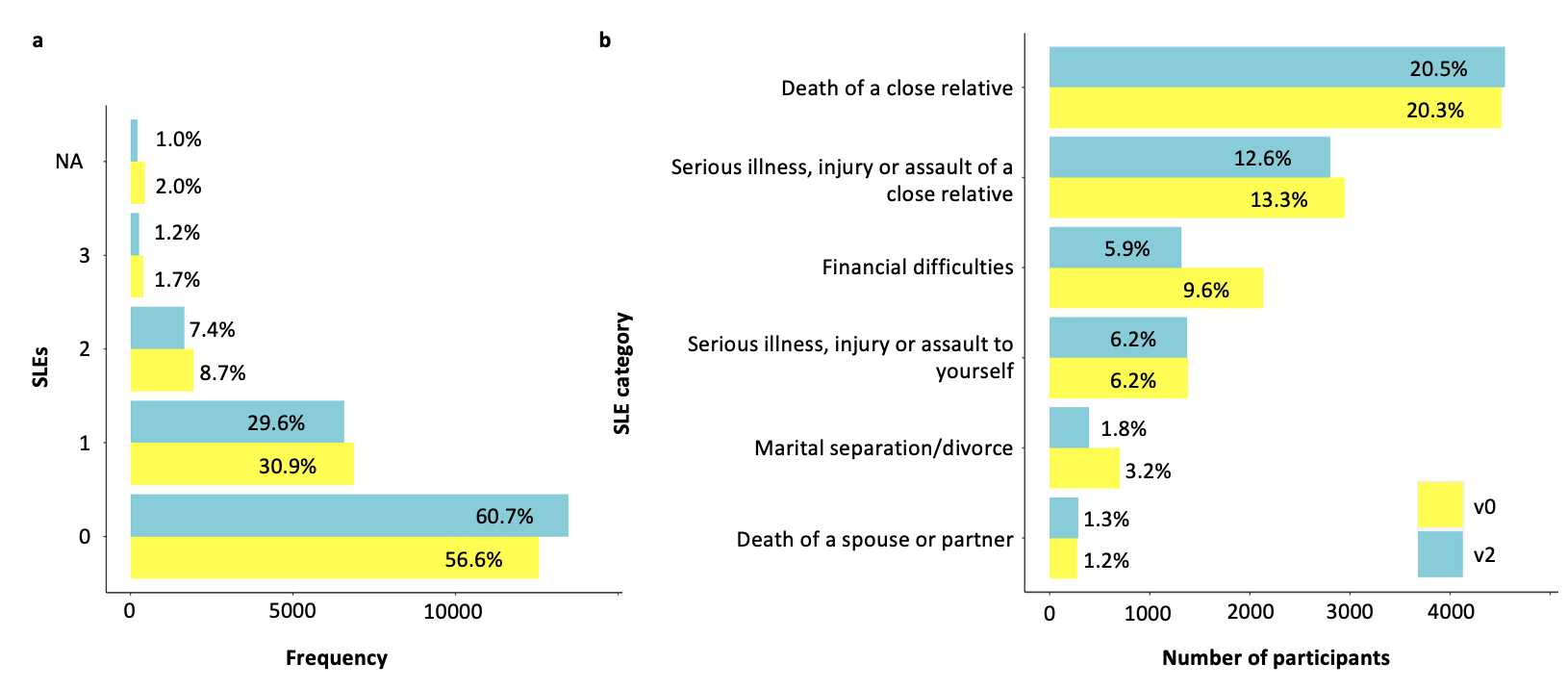
**

Figure S2


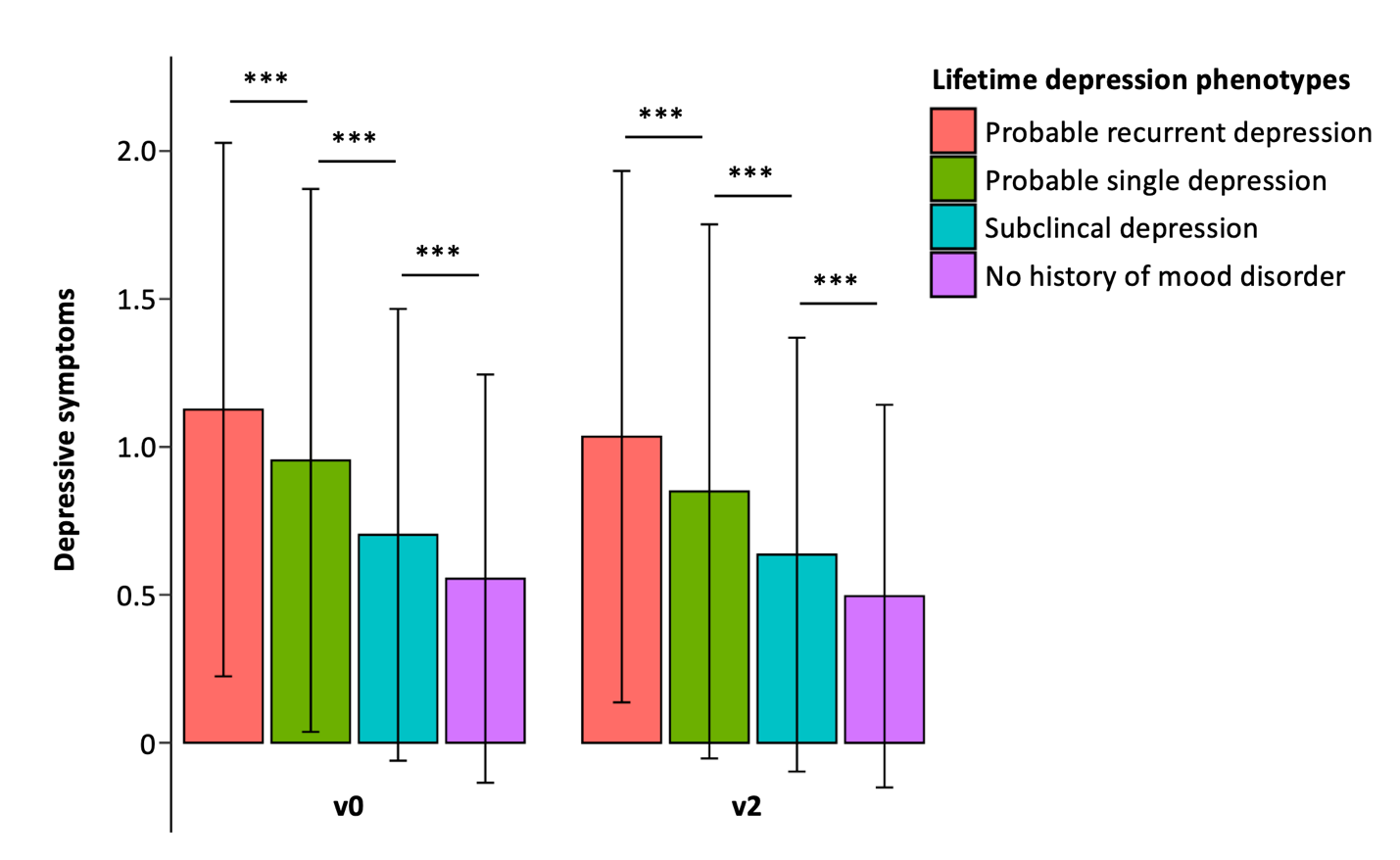


Figure S3


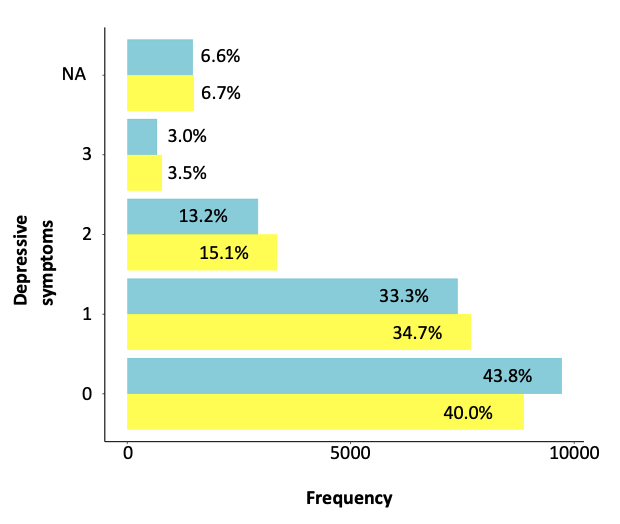
Figure S4


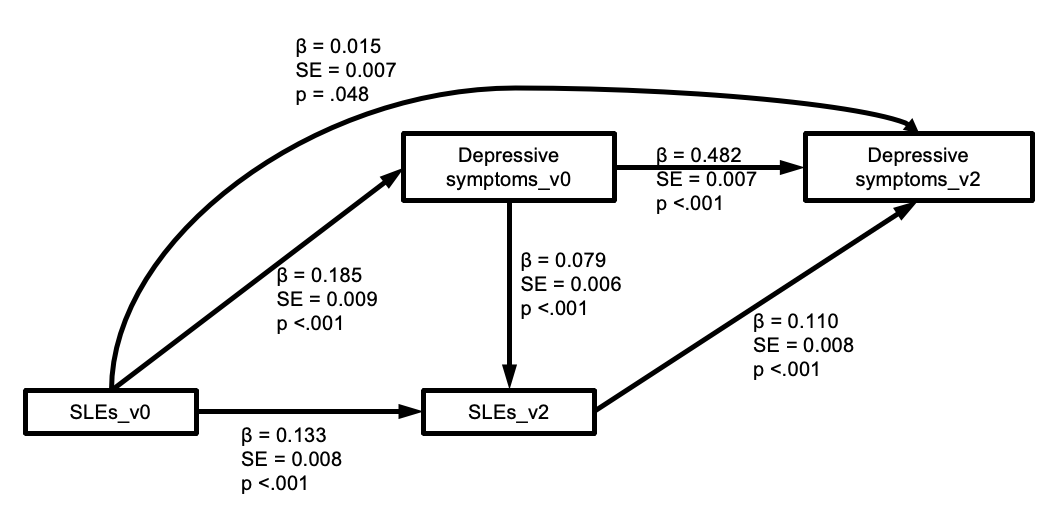

Supplement: supplement [file NIHMS1988932-supplement-supplement.zip › Ma 2024 Psych Med Supp/S0033291723002866sup001.docx]
